# A simple hand‐held magnet array for efficient and reproducible SABRE hyperpolarisation using manual sample shaking

**DOI:** 10.1002/mrc.4687

**Published:** 2018-01-03

**Authors:** Peter M. Richardson, Scott Jackson, Andrew J. Parrott, Alison Nordon, Simon B. Duckett, Meghan E. Halse

**Affiliations:** ^1^ Centre for Hyperpolarisation in Magnetic Resonance (CHyM), Department of Chemistry University of York York UK; ^2^ WestCHEM, Department of Pure and Applied Chemistry and CPACT University of Strathclyde Glasgow UK

**Keywords:** ^1^H, benchtop NMR, Halbach array, hyperpolarisation, NMR, parahydrogen, polarisation transfer field, signal amplification by reversible exchange (SABRE)

## Abstract

Signal amplification by reversible exchange (SABRE) is a hyperpolarisation technique that catalytically transfers nuclear polarisation from parahydrogen, the singlet nuclear isomer of H_2_, to a substrate in solution. The SABRE exchange reaction is carried out in a polarisation transfer field (PTF) of tens of gauss before transfer to a stronger magnetic field for nuclear magnetic resonance (NMR) detection. In the simplest implementation, polarisation transfer is achieved by shaking the sample in the stray field of a superconducting NMR magnet. Although convenient, this method suffers from limited reproducibility and cannot be used with NMR spectrometers that do not have appreciable stray fields, such as benchtop instruments. Here, we use a simple hand‐held permanent magnet array to provide the necessary PTF during sample shaking. We find that the use of this array provides a 25% increase in SABRE enhancement over the stray field approach, while also providing improved reproducibility. Arrays with a range of PTFs were tested, and the PTF‐dependent SABRE enhancements were found to be in excellent agreement with comparable experiments carried out using an automated flow system where an electromagnet is used to generate the PTF. We anticipate that this approach will improve the efficiency and reproducibility of SABRE experiments carried out using manual shaking and will be particularly useful for benchtop NMR, where a suitable stray field is not readily accessible. The ability to construct arrays with a range of PTFs will also enable the rapid optimisation of SABRE enhancement as function of PTF for new substrate and catalyst systems.

## 1 INTRODUCTION

The use of hyperpolarisation for sensitivity enhancement through the generation of non‐equilibrium nuclear spin populations is an increasingly important area of development in magnetic resonance due to its potential to enable new applications in solid‐ and liquid‐state nuclear magnetic resonance (NMR) spectroscopy and magnetic resonance imaging (MRI).^1^, ^2^, ^3^, ^4^, ^5^, ^6^ Of the range of available hyperpolarisation techniques, we focus here on the signal amplification by reversible exchange (SABRE) approach, which is a catalytic method for transferring spin order from the nuclear singlet isomer of H_2_, parahydrogen (*p*‐H_2_), to NMR‐active nuclei in a molecule of interest.^7^ This method is attractive as a hyperpolarisation solution for a number of reasons. First, the hyperpolarisation can be generated quickly (in tens of seconds) and is renewable upon supply of fresh *p*‐H_2_. Second, the source of hyperpolarisation, *p‐*H_2_, is relatively inexpensive to produce and can be stored for weeks to months at room temperature. Third, the level of polarisation that can be achieved (as much as 50% for ^1^H nuclei^8^) is independent of the NMR or MRI detection field. This means that SABRE is a particularly attractive method for sensitivity enhancement of low‐cost and portable benchtop NMR and MRI devices where the detection fields are typically limited to 1–2 T.^9^ Finally, the implementation of a SABRE experiment is relatively straight‐forward, fast, and not technologically demanding compared to other hyperpolarisation methods such as dissolution dynamic nuclear polarisation.^4^

**Figure 1:**
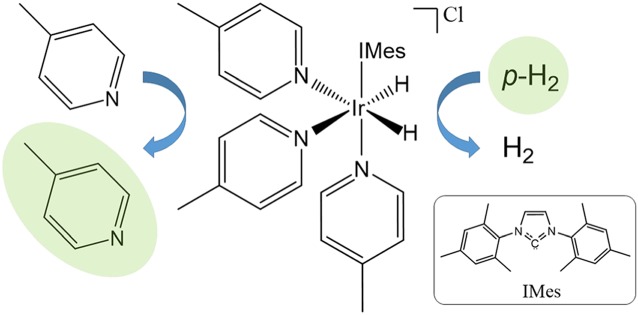
The active form of the signal amplification by reversible exchange polarisation transfer catalyst, [Ir(H)_2_(S)_3_(IMes)]Cl, reversibly binds *p*‐H_2_ and the substrate (*S* = 4‐methylpyridine) promoting catalytic transfer of polarisation from *p*‐H_2_ to the substrate in free solution. Hyperpolarisation is illustrated schematically by the green highlights

Figure 1 presents a schematic illustration of the catalytic SABRE process. In the standard approach, the active SABRE catalyst is a transition metal dihydride complex that binds three molecules of the substrate—two that are oriented trans to the two hydride ligands and one that is oriented trans to a stabilising ligand, typically a *N*‐heterocyclic carbene.^10^ Importantly, the hydrides and the substrate molecules bound trans to the hydrides are in reversible exchange with *p*‐H_2_ and substrate molecules in free solution. When a molecule of *p*‐H_2_ oxidatively adds to the complex, it forms a *J* coupling network with the NMR‐active nuclei on the bound substrate molecules. Under the correct conditions of coupling constants and polarisation transfer field (PTF), this coupling network facilitates the flow of spin‐order from the former *p*‐H_2_‐nuclei to the NMR‐active nuclei on the substrate over a period of a few tens to hundreds of milliseconds. Thus, the bound substrate molecules become hyperpolarised. Because the hydrides and bound substrate molecules are in reversible exchange with free *p*‐H_2_ and free substrate in solution, this process results in a net catalytic transfer of polarisation from the *p*‐H_2_ to the substrate in free solution over a period of seconds. As long as fresh *p*‐H_2_ is supplied, the hyperpolarisation level of the free substrate will build until a steady‐state is reached where the loss of hyperpolarisation through NMR relaxation balances the build‐up of fresh hyperpolarisation through transfer from *p*‐H_2_. Once this steady‐state is reached, the sample is transported into the NMR or MRI instrument for detection.

In order for SABRE to work efficiently and without radio‐frequency intervention, there needs to be strong coupling between the hydrides and the substrate nuclei. Specifically, there exists a resonance condition for optimal transfer of polarisation, whereby the difference in chemical shift between the hydrides and the substrate nuclei is equal to the dominant *J* coupling constant in the network, which is typically the hydride–hydride coupling of the order of 10 Hz.^11^, ^12^, ^13^, ^14^ This resonance condition can be met by carrying out the chemical exchange reaction in a weak PTF prior to NMR or MRI detection at higher magnetic field (typically ≥1 T). The value of the ideal PTF will vary based on the substrate and the identity of the active SABRE catalyst. For homonuclear transfer of polarisation from *p*‐H_2_ to protons on aromatic substrates, the optimum is around *PTF* = 65 G (6.5 mT).

In the simplest implementation of the SABRE technique, the exchange reaction is carried out within an NMR tube that contains a solution of the SABRE catalyst and the substrate of interest under a pressure of *p*‐H_2_‐enriched H_2_ gas. The tube is vigorously shaken in the PTF for a few seconds, to promote dissolution of the *p*‐H_2_ and thus generate a build‐up of SABRE hyperpolarisation on the substrate in free solution. The tube is then manually transferred into an NMR spectrometer for detection. If the detection is carried out using a standard laboratory NMR spectrometer, the PTF is typically supplied by the stray field of the superconducting NMR magnet. Although appealingly simple, this method suffers from a number of draw‐backs. The stray field of the superconducting magnet is highly inhomogeneous, and therefore, it is difficult to reliably and reproducibly shake the NMR tube exclusively in the desired PTF. Furthermore, modern NMR magnets are highly shielded meaning there may not be a convenient region of the stray field where the correct PTF can be accessed. This problem is even more significant when SABRE is implemented with a benchtop NMR spectrometer, where there is no appreciable stray field at all. Several approaches have been introduced that use an electromagnet to generate the PTF.^15^, ^16^, ^17^, ^18^, ^19^, ^20^ In these approaches, *p*‐H_2_ is bubbled through the SABRE solution within an electromagnet, which provides the required PTF (in the range from μT to mT), and then the sample is transported to the NMR spectrometer, either manually or under flow, for signal detection. We note that it has also been demonstrated that SABRE hyperpolarisation can be detected in the low‐field (μT to mT) regime where no transport of the sample is required.^18^, ^21^ The use of an electromagnet to generate the PTF is advantageous in terms of reproducibility and hyperpolarisation optimisation as it provides software control over the SABRE polarisation time and the PTF. Furthermore, in the case of the automated flow approach, the transfer time between the polarisation and detection stages of the experiment is also well‐controlled.^15^, ^16^ However, the equipment required for the bubbling of *p*‐H_2_ and the electromagnet adds a layer of cost and complexity to the SABRE experiment that may not be desirable for all applications. In addition, the levels of polarisation observed using an automated flow system are often found to be much less than those achieved using the manual shaking approach.^15^ This may be due to a combination of inefficient *p*‐H_2_ mixing during the bubbling step, when compared to manual shaking, the lower level of *p*‐H_2_ enrichment in the gas used for bubbling and the increased transfer time in the automated flow approach, during which the hyperpolarisation will decay due to NMR relaxation.

In this work, we present an alternative, simple, and cost‐effective solution to generating a constant PTF for SABRE experiments: a hand‐held magnet array for manual shaking of the SABRE sample. Our hand‐held device consists of solid‐state magnets arranged in a Halbach design^22^ to generate a relatively homogeneous field transverse to the long axis of a cylinder into which the NMR sample is placed. The entire unit, consisting of the NMR tube and magnet array, is manually shaken to allow for the SABRE transfer to take place within the desired PTF prior to transfer of the sample into the NMR spectrometer for detection. This method ensures the reproducibility of the PTF during manual SABRE experiments, and by making small changes to the magnet array design, a range of PTFs can be generated allowing for the optimisation of SABRE polarisation transfer using the manual approach.

## 2 RESULTS AND DISCUSSION

### 2.1 Hand‐held magnet array design

**Figure 2:**
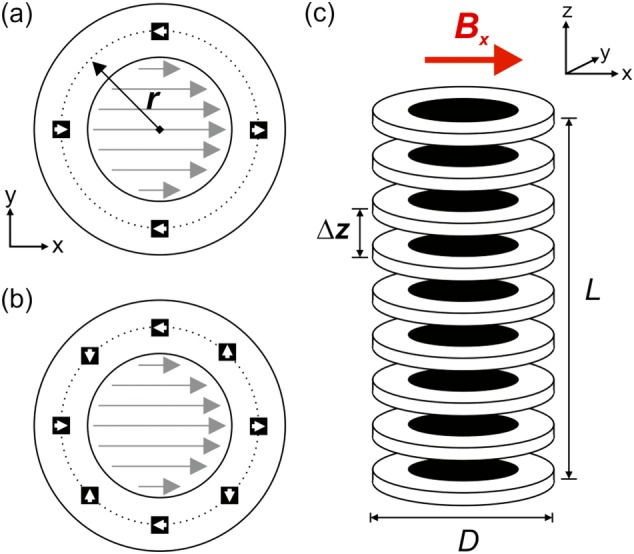
Schematic of the hand‐held signal amplification by reversible exchange magnet array. Each individual ring is composed of (a) 4 or (b) 8 solid‐state magnets fixed at a distance *r* from the centre of the ring (dashed line). The direction of polarisation of these individual magnets is arranged into a Halbach configuration in order to generate a homogeneous field along *x* in the centre of the ring. (c) A set of *N* rings is combined together with a uniform spacing of Δ*z* to form a cylinder of length *L* with an outer diameter of *D*. A sample within an NMR tube, placed into the centre of the cylinder, will experience a net magnetic field, *B*_*x*_, transverse to the long (*z*) axis of the cylinder

Our hand‐held magnet array is based on a Halbach design.^22^ We start with a ring where *n* = 4 magnets are placed at a fixed distance from the centre, *r*, and with the direction of polarisation of each magnet arranged as shown in Figure 2a. This arrangement roughly mimics the field lines from a magnetic dipole and thus generates a constant transverse field in the centre of the ring, *B*_*x*_. The magnitude of the field generated will depend on the size and type of magnets used and the radius, *r*, of the ring. In order to generate a field that extends along the length of an NMR tube, a series of *N* rings are combined together, with a fixed separation between the centre of two adjacent rings of Δ*z*, to form a cylinder of length *L* with an outer diameter of *D* (Figure 2c). The net magnetic field along the long (*z*) axis of the cylinder will be the sum of the overlapping fields from the individual rings. Therefore, the magnitude and homogeneity of the field generated, *B*_*x*_, can be controlled by the choice of the magnet ring radius, *r*, and the ring separation, Δ*z*.

**Figure 3:**
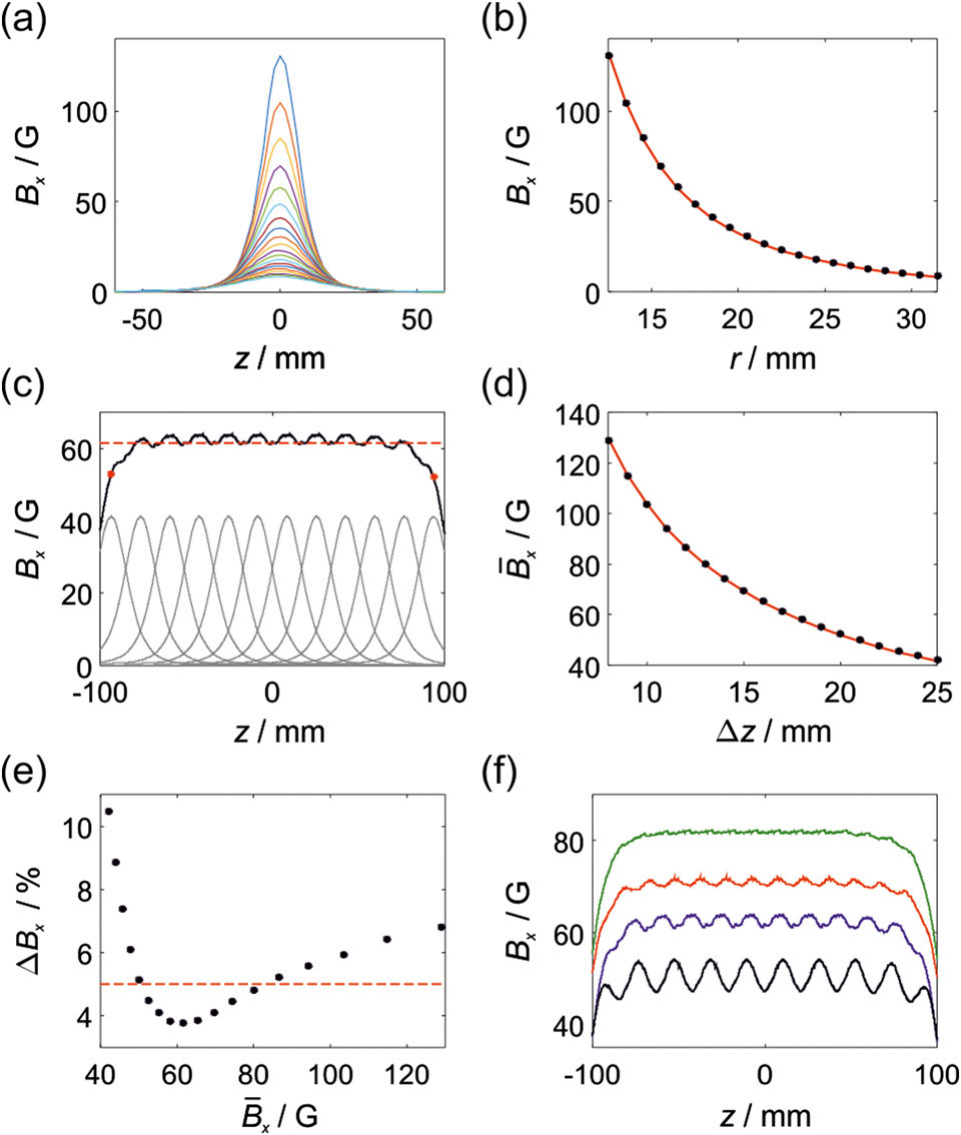
(a) Transverse field (*B*_*x*_) along the *z* axis of a single Halbach ring with *r* ranging from 12.5 to 31.5 mm. (b) Transverse field, *B*_*x*_, at the centre of a single ring as a function of *r*. Red line is a fit to *B*_*x*_(0) = *A*_0_*r*^−3^ with *A*_0_ = 2.582 × 10^5^ G ⋅ mm^3^. (c) Total field, *B*_*x*_, along the central z‐axis of a cylinder consisting of *N* = 12 rings with *r* = 18.5 mm separated by Δ*z* = 17 mm. The total field is calculated as the sum of the overlapping profiles of the individual rings (grey). The average field in the centre of the cylinder is ${\bar{B}}_{x}=62.5\;G$ (dashed red line). (d) Average transverse field for a cylinder with rings with *r* = 18.5 mm as a function of ring separation, Δ*z*. Red line is ${\bar{B}}_{x}=\left(1039\;G\cdot \mathrm{mm}\right)\;{\left(\Delta ,z\right)}^{-1}$. (e) Standard deviation (% relative to the mean) of the total field along the length of the cylinder for different ring separations (*r* = 18.5 mm) and hence different total fields. Dashed line indicates a 5% standard deviation. (f) Total field profiles for cylinders made up of rings with *r* = 18.5 mm and Δ*z* = 21 mm (black, ${\bar{B}}_{x}=50.0\;G$), Δ*z* = 17 mm (blue, ${\bar{B}}_{x}=61.5\;G$), Δ*z* = 15 mm (red, ${\bar{B}}_{x}=69.6\;G$), and Δ*z* = 13 mm (green, ${\bar{B}}_{x}=80.0\;G$)

Halbach arrays have been used extensively to generate homogeneous *B*_0_ fields for NMR and MRI applications,^23^, ^24^ and many sophisticated methods have been developed to simulate and optimise the fields from permanent magnet arrays to the necessary level of precision to support NMR spectroscopy.^25^, ^26^ For the proposed SABRE application, our aim is to design magnet arrays with a field variation along the length of the centre of the cylinder of better than ~5%. Given this nonstringent homogeneity requirement, we have chosen to use a simple empirical approach to modelling the associated magnetic fields. In the first step, a series of rings were constructed from 3D printed templates with four rectangular magnets (2.5 mm × 7.5 mm × 2.5 mm N42 grade nickel‐coated NbFeB) placed at radii ranging from *r* = 12.5 mm to *r* = 31.5 mm, according to Figure 2a. The field, *B*_*x*_, of each magnet array was measured at the centre of the *xy* plane as a function of distance from the ring along *z*, where *z* = 0 corresponds to the middle of the magnet array. Example magnetic field profiles are shown in Figure 3a, and the field at the centre of each ring (*z* = 0) is plotted as function of *r* in Figure 3b. The field demonstrates a *r*^−3^ dependence (red line in Figure 3b). The constant of proportionality in our case was found to be *A*_0_ = 2.582 × 10^5^ G ⋅ mm^3^.

The field from a full cylinder was calculated as the sum of overlapping ring profiles for a given magnetic ring radius, *r*, and separation, Δ*z*, as illustrated in Figure 3c for *r* = 18.5 mm, *N* = 12, and Δ*z* = 17 mm. This configuration gives rise to a cylinder with an average field of ${\bar{B}}_{x}=61.5\;G$ (dashed red line), calculated as the mean of the field *B*_*x*_ along the length of the cylinder from the centre of the first ring to the centre of the final ring (indicated by the red dots in Figure 3c). The average field can be controlled by changing the ring separation, as shown in Figure 3d for a cylinder constructed from magnet arrays with *r* = 18.5 mm and ring spacings varying from Δ*z* = 8 to 25 mm. The average field was found to be proportional to the inverse of the ring spacing (red line in Figure 3d), with the constant of proportionality between the average field of the cylinder and the ring spacing found to depend on the inverse square of the magnet array radius. Therefore, the average field of a cylinder as a function of both *r* and Δ*z* was modelled as ${\bar{B}}_{x}=C_{0}\;r^{-2}\Delta z^{-1}$ with a constant of proportionality for our design of *C*_0_ = 3.55 × 10^6^ G ⋅ mm^3^.

In order to design an effective SABRE magnet array, we also need to consider the field homogeneity along the axis of the cylinder. For example, the level of field inhomogeneity, calculated as the standard deviation of *B*_*x*_ over the length of the cylinder, was found to be Δ*B*_*x*_ = 3.8% for the example in Figure 3c. Inspection of the plot of the total field (black line) reveals two sources of field inhomogeneity. First, an oscillation in the maximum value of the field that comes from imperfect overlap of the magnetic field profiles from the individual rings. This can be minimised by decreasing the separation between adjacent rings, Δ*z*. The second source of inhomogeneity is the fall‐off of the field at the ends due to the finite length of the cylinder. The extent of this fall‐off region will be increased by decreasing the separation between the rings. Therefore, the relationship between ring separation and field homogeneity will be a compromise between these two effects. This is illustrated by the plot of field inhomogeneity as a function of average magnetic field presented in Figure 3e for the case of *r* = 18.5 mm, where the different magnetic fields correspond to different values of Δ*z* according to Figure 3d. The inhomogeneity increases dramatically at both lower magnetic field (large Δ*z*) and higher magnetic field (small Δ*z*). Applying a limit of 5% inhomogeneity (dashed red line), we find that using a fixed ring diameter of *r* = 18.5 mm, cylinders with average magnetic fields between *B*_*x*_ = 50 – 80 G can be constructed with field inhomogeneity of approximately 5% or less. The predicted magnetic fields for these cylinders (Figure 3f) illustrate the trade‐off between the large Δ*z* case (50 G, black), which has a large field oscillations along the axis, and the small Δ*z* case (80 G, green), which has a more homogeneous region in the middle of the cylinder but a more severe drop off at the ends. We note that this latter issue could be mitigated by making the cylinder much longer than the NMR sample; however, this is not an ideal solution as it will make the shaking of the sample more cumbersome.

In order to construct cylinders with fields weaker than 50 G and with acceptable field homogeneity, magnet arrays with a larger radius will be required. However, cylinders with magnetic fields stronger than 80 G of acceptable homogeneity can be obtained in two ways. First, magnet arrays with smaller *r* could be used. Alternatively, the magnet arrays could be constructed using *n* = 8 magnets, as illustrated in Figure 2b. By doubling the number of magnets in the ring, the net magnetic field produced will be approximately doubled. In addition, it is anticipated that, within the ring, the homogeneity of the field will be improved by using a more complete Halbach array. Therefore, *n* = 8 magnet arrays with *r* = 18.5 mm could be used to construct cylinders with an average field ranging from ${\bar{B}}_{x}=100-160\;G$.

### 2.2 Hand‐held SABRE magnet array implementation

**Figure 4:**
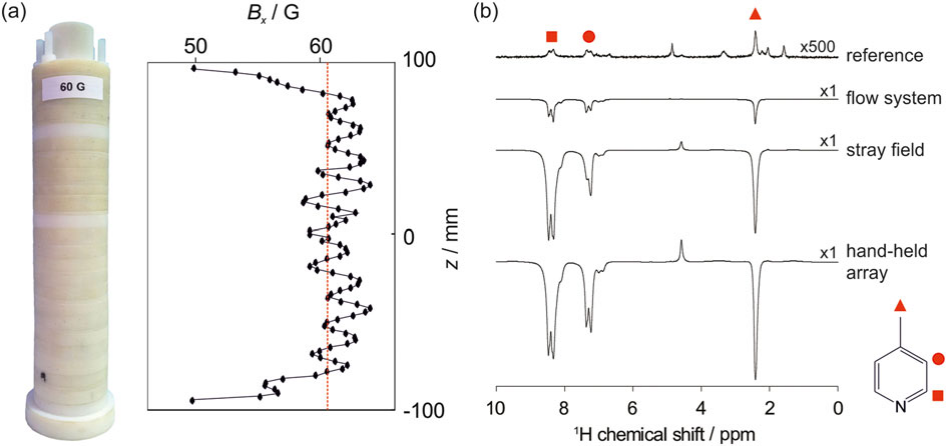
(a) Photo of the hand‐held array and corresponding magnetic field profile measured along the central axis of the cylinder. The mean field, ${\bar{B}}_{x}=60.6\;G$ is indicated by the dashed red line. (b) Comparison of hyperpolarised 43 MHz benchtop ^1^H nuclear magnetic resonance (NMR) spectra of 52 mM 4‐methylpyridine (with 5.2 mM signal amplification by reversible exchange catalyst in *d*_4_‐methanol) acquired using: an automated flow system with 15 s bubbling of *p*‐H_2_ through the solution within an electromagnet and 4 s of manual shaking of an NMR tube under 4 bar *p*‐H_2_ in the stray field of a 400 MHz spectrometer and in the hand‐held magnet array shown in (a). A reference thermally polarised ^1^H NMR spectrum is included for comparison

To test the hand‐held SABRE magnet array design, a 3D printer was used to generate templates for a cylinder with a target field of 61.5 G, that is, near the typical optimal PTF for SABRE hyperpolarisation of ^1^H nuclei. The cylinder was made up of *N* = 12 rings, each containing *n* = 4 magnets placed at a radius of *r* = 18.5 mm. These rings were combined at a separation of Δ*z* = 17 mm to form a cylinder of *L* = 187 mm and *D* = 47 mm. A photo of the completed cylinder and the corresponding magnetic field profile measured along the central axis of the array are presented in Figure 4a. The measured average field, 60.7 G, is in good agreement with the predicted field of 61.5 G, whereas the inhomogeneity of the constructed cylinder (4.6%) is slightly higher than the predicted inhomogeneity (3.8%).

To evaluate the functionality of the hand‐held magnet array, a series of SABRE hyperpolarisation measurements were carried out on a sample containing 52 mM of substrate (4‐methylpyridine) and 5.2 mM of SABRE catalyst in *d*_4_‐methanol. In all cases, ^1^H NMR spectra were acquired on a 1 T benchtop NMR spectrometer (Magritek). The SABRE experiment was carried out in three ways. First, an automated flow system, described previously,^15^ was used to bubble *p*‐H_2_ through the catalyst/substrate solution (3 ml) for 15 s within a PTF of 60 G generated by an electromagnet before flowing the sample under a pressure of N_2_ gas into the NMR spectrometer for signal detection. Second, an NMR tube containing the substrate/catalyst solution (0.6 ml) under a pressure of 4 bar *p*‐H_2_ was shaken in the stray field of a 9.4 T superconducting NMR magnet at ~60 G for 4 s before manual transfer to the NMR spectrometer for detection. Finally, an NMR tube containing the substrate/catalyst solution (0.6 ml) under a pressure of 4 bar *p*‐H_2_ was placed into the hand‐held magnet array and shaken for 4 s prior to manual transfer of the sample to the NMR spectrometer for signal detection. Each method was repeated 10 times to assess the reproducibility of the SABRE response. Example SABRE‐enhanced ^1^H NMR spectra acquired with the three methods are presented in Figure 4b along with a standard ^1^H NMR spectrum (acquired without hyperpolarisation) for reference. The average SABRE enhancement factor, polarisation level, and reproducibility for the three methods are summarised in Table 1. The reproducibility was calculated as the standard deviation in observed enhancement factor over the 10 repetitions expressed as a percentage of the average enhancement factor.

**Table 1:** Average SABRE enhancement factor, polarisation level, and standard deviation for the three distinguishable ^1^H resonances of 4‐methylpyridine (ortho, meta, and methyl) calculated from 10 repeat measurements using three different methods of generating the polarisation transfer field (PTF)

SABRE method		Average enhancement factor	Average polarisation level (%)	Standard deviation (%)
Flow system (electromagnet)	Ortho	−1,263	0.441	4.5
	Meta	−646	0.226	4.7
	Methyl	−392	0.137	4.6
	Total	−713	0.249	4.6
Manual shaking (stray field)	Ortho	−6,437	−2.25	10.2
	Meta	−3,110	−1.087	15.3
	Methyl	−1,802	−0.630	9.1
	Total	−3,500	−1.22	9.1
Manual shaking (hand‐held array)	Ortho	−7,740	−2.70	5.0
	Meta	−4,777	−1.67	5.4
	Methyl	−2,076	−0.726	21.4
	Total	−4,466	−1.56	5.8

*Note*. *PTF*~60 G in all cases. The total enhancement was calculated from the sum of the integrals of all substrate resonances in the ^1^H NMR spectra. SABRE = signal amplification by reversible exchange.

One of the primary benefits of the hand‐held magnet array is an increase in the observed SABRE enhancement compared to the other two methods. Use of the hand‐held array yields a 600% increase in total polarisation over the automated flow approach and a 25% increase over the stray‐field shaking method. The increase in SABRE enhancement factor between the hand‐held array and stray field approaches arises from a combination of the improved PTF homogeneity experienced by the nuclei during the exchange reaction and a reduction in sample transfer times in the hand‐held array case, as the shaking is carried out adjacent to the benchtop spectrometer rather than adjacent to a neighbouring high‐field NMR spectrometer. The largest increase in SABRE enhancement (50%) is observed for the substrate meta protons. This is to be expected because the phase of the SABRE enhancement of the meta proton resonance is highly dependent on the magnitude of the PTF. Therefore, the efficiency of hyperpolarisation is expected to benefit significantly from the increased PTF homogeneity provided by the hand‐held magnet array approach.

The second benefit of the hand‐held magnet array is an increase in reproducibility between repeat measurements when compared to the stray field method. As illustrated in Table 1, the best reproducibility is achieved by the flow system (4.6%), followed by the hand‐held array (5.8%), and then the stray field approach (9.1%). The flow system is the most reproducible because it provides a high level of control over both the exact PTF experienced by the sample during SABRE and the sample transfer time. The hand‐held shaker provides control over the PTF during shaking but not the subsequent transfer to the detector. The importance of a consistent sample transfer time can be seen through the enhancement factor of the methyl resonance. At very short transfer times (<2 s), this resonance displays some antiphase character, likely due to the hyperpolarisation of a fast relaxing multispin‐order term. Therefore, for rapid sample transfer times the total SABRE enhancement factor, calculated as the integral of the entire resonance, can appear reduced. The extent to which the enhancement factor is reduced will be highly dependent on the exact transfer time. The hand‐held magnet array measurements are particularly susceptible to this effect due to the short transfer times. Indeed, the poor reproducibility for the methyl resonance in the hand‐held array measurements (21.4%) is largely derived from 2 of the 10 measurements where the methyl resonance contained a significant antiphase component.

In addition to allowing for manual SABRE to be performed in the absence of a suitable stray field and improving efficiency and reproducibility, the hand‐held magnet array approach also allows for the investigation of the variation in SABRE enhancement as a function of PTF using the manual shaking approach. In order to demonstrate this, a range of hand‐held magnet arrays were designed and constructed with PTFs ranging from 30 to 140 G. The features of these arrays are summarised in Table 2. Photos of the arrays and plots of their magnetic field profiles are available in Supporting Information. The measured average field, ${\bar{B}}_{x,\exp}$, is in good agreement with the average field predicted from our empirical modelling approach, ${\bar{B}}_{x,\text{pred}}$, in all cases (<3.5% deviation), whereas the observed field inhomogeneity is slightly increased. This comes from the variability in the strength and polarisation of the individual magnets as well as variations in the thickness of the rings and separators. Nevertheless, the field homogeneity for all eight magnet arrays lies within an acceptable range of 4.6–5.6%.

**Table 2:** Summary of the design parameters for eight hand‐held magnet arrays including the predicted and measured average field strength, ${\bar{B}}_{x}$, and standard deviation of the field, Δ*B*_*x*_ along the centre of the cylinder from the centre of the first ring to the centre of the *N*th ring

#	*r* (mm)	Δ*z* (mm)	*n*	*N*	D (mm)	*L* (mm)	${\bar{B}}_{x,\text{pred}}$ (G)	Δ***B***_***x***, ***pred***_ (%)	${\bar{B}}_{x,\text{exp}}$ (G)	Δ***B***_***x***, ***exp***_ (%)
1	25.5	17	4	12	61	187	30.9	5.4	31.1	5.2
2	18.5	21	4	10	47	189	50.0	5.1	49.9	5.2
3	18.5	17	4	12	47	187	61.5	3.8	60.6	4.6
4	18.5	15	4	14	47	195	69.6	4.1	68.8	4.9
5	18.5	13	4	16	47	195	80.0	4.8	81.0	5.3
6	18.5	21	8	10	47	189	100.0	5.1	96.7	5.6
7	18.5	17	8	13	47	204	123.2	3.7	120.9	5.3
8	18.5	15	8	13	47	180	138.9	4.2	135.4	5.2

**Figure 5:**
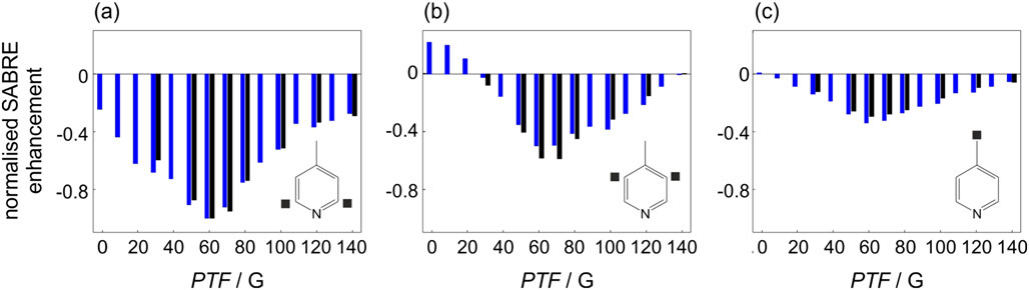
Normalised signal amplification by reversible exchange (SABRE) enhancement factor as a function of polarisation transfer field for the three ^1^H resonances of 4‐methylpyridine: (a) ortho, (b) meta, and (c) methyl. SABRE experiments carried out by bubbling *p*‐H_2_ at a pressure of 4 bar through the solution within an electromagnet are shown in blue and those obtained by manually shaking an NMR tube containing the solution under 4 bar *p*‐H_2_ within a hand‐held magnet array (average of three measurements) are in black. All SABRE enhancement factors are normalised to the maximum ortho ^1^H enhancement factor at PTF = 60 G for the given method

The performance of SABRE carried out using hand‐held arrays with different PTFs was assessed by measuring the SABRE enhancement of the three ^1^H resonances of 4‐methylpyridine as a function of PTF and comparing these results to SABRE experiments carried out using the flow system, where the PTF is generated by an electromagnet under software control (Figure 5). As demonstrated previously, the absolute SABRE enhancement obtained using the hand‐held arrays is much greater than for the automated flow system. Therefore, Figure 5 presents a comparison of the relative SABRE enhancement, where each measurement has been normalised to the maximum enhancement observed for the ortho proton of 4‐methylpyridine in a PTF of 60 G. Figure 5 shows that the PTF dependence of the SABRE enhancement obtained using the hand‐held shakers (black) follows the same trend as for the electromagnet in the flow system (blue), with excellent agreement across all three of the different ^1^H resonances within the substrate. This suggests that the levels of field homogeneity achieved in the hand‐held shakers are not limiting the effectiveness of the SABRE polarisation transfer for this particular substrate and that the hand‐held magnet arrays can be used to probe the PTF‐dependence of SABRE.

## 3 EXPERIMENTAL

The hand‐held magnet arrays were constructed using rectangular N42 nickel‐plated NdFeB 2.5 mm × 7.5 mm × 2.5 mm magnets (http://first4magnets.com). The magnets (*n* = 4 or 8, according to Table 2) were placed into formers manufactured either by 3D printing (Cylinder 3) or laser cutting of 8 mm thick Perspex sheets (Cylinders 1–2 and 4–8) and arranged according to Figure 2a. In all cases, the internal diameter of each ring was 27 mm and included a notch to aid alignment. *N* magnet‐containing rings were combined with magnet‐free rings that acted as spacers to form a cylinder, as shown in Figure 2c, where the fixed distance between the middle of adjacent magnet‐containing rings was Δ*z*. The rings were aligned and held together by four threaded 5‐mm nylon rods that were screwed into a solid 10‐mm‐thick base and fixed in place by four nylon nuts at the top of the cylinder. The total length of the resultant arrays (*L*), from the middle of the first ring to the middle of the final ring, is given in Table 2. An additional 3D‐printed insert was used to hold the NMR sample within the centre of the array during shaking. The placement of this insert was designed such that the bottom of the NMR tube was held ~ 5 mm from the bottom of the array, and the NMR tube was fixed relative to the array during shaking. The magnetic field profiles along the central axis of the final arrays were measured using a Hirst Magnetics GM08 hand‐held gaussmeter with a transverse probe. The magnet array field predictions were carried out using home‐written code in MATLAB, and all curve fitting was carried out using the MATLAB curve fitting toolbox.

All NMR samples contained 5.2 mM of the SABRE precatalyst Ir [(COD) (IMes) (Cl)] (where COD = 1,5‐cyclooctadiene and IMes = 1, 3‐bis (2,4,6‐trimethylphenyl)‐imidazolium) with 52 mM of the substrate (*S* = 4‐methylpyridine). The catalyst and substrate were added to either 0.6 ml (for manual shaking experiments) or 3 ml (for flow experiments) of *d*_4_‐methanol and mixed until fully miscible. The manual shaking samples were then loaded into a NMR tube fitted with a Young's tap and degassed using a freeze‐pump‐thaw procedure in an acetone bath using dry ice. The flow samples were placed directly into the mixing chamber of the flow system and subsequently subjected to a nitrogen atmosphere. Activation, that is, conversion of the SABRE precatalyst to the active form: [Ir(H)_2_(4‐methylpyridine)_3_(IMes)]Cl, was achieved by repeated exposure of the sample to fresh *p*‐H_2_‐enriched H_2_ gas by either charging the NMR tube with a pressure of 4 bar of *p*‐H_2_ and shaking vigorously or by bubbling *p*‐H_2_ through the solution within the mixing chamber of the flow system. To ensure complete conversion of the SABRE precatalyst to the active form, this procedure was repeated at least 7 times over a period of 10 min, with fresh *p*‐H_2_ being added to the headspace of the NMR tube between each repetition. Following activation, the sample was left to equilibrate within the benchtop NMR spectrometer for 5 min before a single scan, thermally‐polarised reference ^1^H NMR spectrum was acquired.

The automated flow system used herein has been described previously in the context of high‐field NMR^15^, and we have recently integrated it with a benchtop NMR spectrometer (1 T Magritek Spinsolve) in a comparable manner.^27^ The sample is loaded into a mixing chamber (*L* = 75.0 mm and *d* = 13.0 mm) that sits within a solenoid coil (*L* = 100 mm and *d* = 27.5 mm). Parahydrogen‐enriched H_2_ gas is bubbled through the solution via a porous frit within the mixing chamber for 15 s at a pressure of 4 bar. Following bubbling, the H_2_ gas pressure is released, and the sample flows into a glass insert within the benchtop NMR spectrometer under a pressure of N_2_ gas. The delay between the cessation of bubbling and the detection of the SABRE hyperpolarisation within the NMR spectrometer is typically between 4 and 5 s. The *p*‐H_2_‐enriched gas was produced by a Bruker *p*‐H_2_ generator operating at a conversion temperature of 38 K to produce ~ 92% *p*‐H_2_ enrichment. The *p*‐H_2_ bubbling time was optimised to give the largest SABRE enhancement.

For the manual shaking SABRE experiments, a homebuilt *p*‐H_2_ generator (described previously^28^) with a conversion temperature of 28 K was used to achieve ~98% *p*‐H_2_ enrichment. Between each manual shaking experiment, the headspace of the NMR tube was evacuated and charged with fresh *p*‐H_2_ at a pressure of 4 bar. For both the stray field and hand‐held magnet array SABRE experiments, the NMR sample was vigorously shaken for 4 s and then manually transferred to the NMR spectrometer for signal detection. We note that the sample will necessarily experience a varying external magnetic field during the manual transfer period. It is assumed that this transfer is adiabatic. No depolarisation effects due to rapidly changing fields during sample transport were observed. Transfer times were typically 2–3 s, with slightly faster transfer times being achieved in the hand‐held magnet array case due to the shorter distance to the benchtop NMR spectrometer. The shaking time was optimised to give the largest observed SABRE enhancement.

All ^1^H NMR spectra were acquired in a single scan on a benchtop NMR spectrometer (Magritek Spinsolve) operating at a ^1^H Larmor frequency of 43.318 MHz using a simple 90° radio‐frequency pulse and detect sequence. SABRE enhancement factors, *ε*, were calculated as the ratio of the integral of a given SABRE‐enhanced ^1^H NMR peak to the integral of a reference ^1^H NMR peak from a thermally‐polarised NMR spectrum. Due to spectral overlap of the meta and methyl ^1^H resonances of the substrate, 4‐methylpyridine, with resonances from the SABRE catalyst, the integral of the ortho ^1^H resonance in the thermally‐polarised NMR spectrum was used to determine the reference signal. The SABRE polarisation level, *P*_*SABRE*_, was calculated from the enhancement factor, *ε*, according to Equation (1), where *γB*_0_ = 2*π* * 43.318 MHz is the ^1^H NMR Larmor frequency, *T* = 298 K is the temperature of the sample within the NMR spectrometer, *k*_*B*_ is Boltzmann's constant, and ℏ is Planck's constant divided by 2*π*.

(1) $$P_{\text{SABRE}}=\varepsilon P_{\text{Boltzman}}=\varepsilon \frac{\gamma B_{0}\hbar }{2k_{B}T}$$

## 4 CONCLUSIONS

In this work, we have presented a method for designing and implementing a hand‐held magnet array for use in SABRE hyperpolarisation experiments using a manual shaking approach. These arrays are based on a Halbach design and can be adapted to achieve polarisation transfer fields in the tens of gauss range. We have demonstrated that the hand‐held arrays provide improved reproducibility and SABRE efficiency over the stray field approach. Furthermore, the ability to generate arrays with a range of average field values allows for the experimental investigation of the PTF‐dependence of SABRE hyperpolarisation without the need to use an automated flow system. We anticipate that these hand‐held arrays will be particularly useful in cases, such as SABRE‐enhanced benchtop NMR, where no appropriate stray field is available to provide the necessary PTF. In the examples shown here, PTFs with homogeneities between 4.6% and 5.6% were achieved and it was found that for the substrate studied, 4‐methylpyridine with SABRE catalyst [Ir(H)_2_(4‐methylpyridine)_3_IMes]Cl in *d*_4_‐methanol, this was sufficient to achieve efficient polarisation transfer and to study the PTF dependence of the SABRE enhancement factor. However, it is possible that other substrate‐catalyst systems may have narrower resonance conditions. In such cases, it would be advantageous to design a more homogeneous magnet array to achieve optimal SABRE enhancement. This can be achieved by modifying our approach to include variable spacing of the magnet rings. In particular, decreased spacing of the outermost rings would increase the magnetic field overlap at the ends of the array and so would counter the fall‐off of the field due to the finite length of the cylinder. In addition to this change in design, the construction method could be improved to minimise its impact on the field homogeneity of the completed array. We found that the largest source of construction‐derived field inhomogeneity arose not from magnet variability but rather from the inconsistency in the thickness of the rings and spacers obtained through laser cutting. Construction of the arrays using careful selection of the laser‐cut pieces based on their measured dimensions or using formers manufactured using other methods (e.g., injection moulding or 3D printing) could further improve field homogeneity.

## Supporting information


**DATA ACCESS STATEMENT**
All experimental NMR data reported in this work is available via Research Data York at http://dx.doi.org/10.15124/cd106de7-23bd-4411-a88e-a6d7f9604cc5

**Figure S1.** Photos of 8 hand‐held magnet arrays
Figure S2. Photos of single magnet rings
Figure S3. Magnetic field profiles for the eight magnet arrays
Table S1. SABRE enhancement factors for repetition measurements
Table S2. SABRE enhancement factors for PTF‐dependent shaking measurements
Table S3. SABRE enhancement factors for PTF‐dependent flow system measurements
