# Supplementary material for: A simple hand‐held magnet array for efficient and reproducible SABRE hyperpolarisation using manual sample shaking
Source: Magn Reson Chem. 2018 Jan 3;56(7):641–50. doi: 10.1002/mrc.4687 (PMC6001426; doi:10.1002/mrc.4687)
Supplement: Supplementary file 1 — Figure S1. Photos of 8 hand‐held magnet arrays Figure S2. Photos of single magnet rings Figure S3. Magnetic field profiles for the eight magnet arrays Table S1. SABRE enhancement factors for repetition measurements Table S2. SABRE enhancement factors for PTF‐dependent shaking measurements Table S3. SABRE enhancement factors for PTF‐dependent flow system measurements [file MRC-56-641-s001.pdf]

Supporting information: A simple hand-held magnet array for efficient and reproducible SABRE hyperpolarisation using manual sample shaking

Peter M. Richardson,[1] Scott Jackson,[1] Andrew Parrott,[2] Alison Nordon,[2] Simon B. Duckett[1] and Meghan E. Halse[1]*

[1]Centre for Hyperpolarisation in Magnetic Resonance (CHyM), Department of Chemistry, University of York, York, UK

[2]Department of Pure and Applied Chemistry, University of Strathclyde, Glasgow, UK

Figure S1. Photos of 8 hand-held magnet arrays 2

Figure S2. Photos of single magnet rings 2

Figure S3. Magnetic field profiles for the eight magnet arrays 2

Table S1. SABRE enhancement factors for repetition measurements 3

Table S2. SABRE enhancement factors for PTF-dependent shaking measurements 4

Table S3. SABRE enhancement factors for PTF-dependent flow system measurements 4

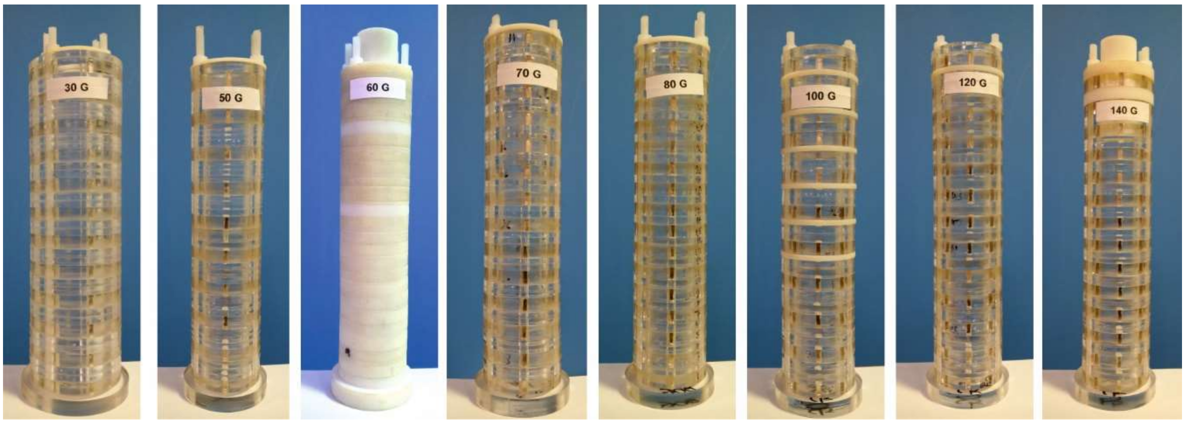

Figure S1 Photos of the 8 hand-held magnet arrays detailed in Table 2. Each cylinder is made up of a combination of rings containing magnets and spacers. The cylinder is held together by four threaded teflon rods (5 mm), which screw into the base and are held in place by four nuts at the top of the array. The inner diameter of all cylinders is 27 mm, with a notch included to ensure correct alignment of each individual ring.

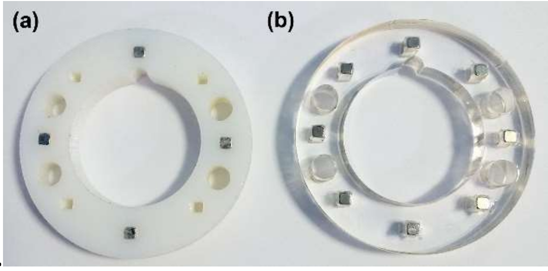

Figure S2 (a) 3D-printed ring containing 4 magnets. (b) Laser-cut ring containing 8 magnets.

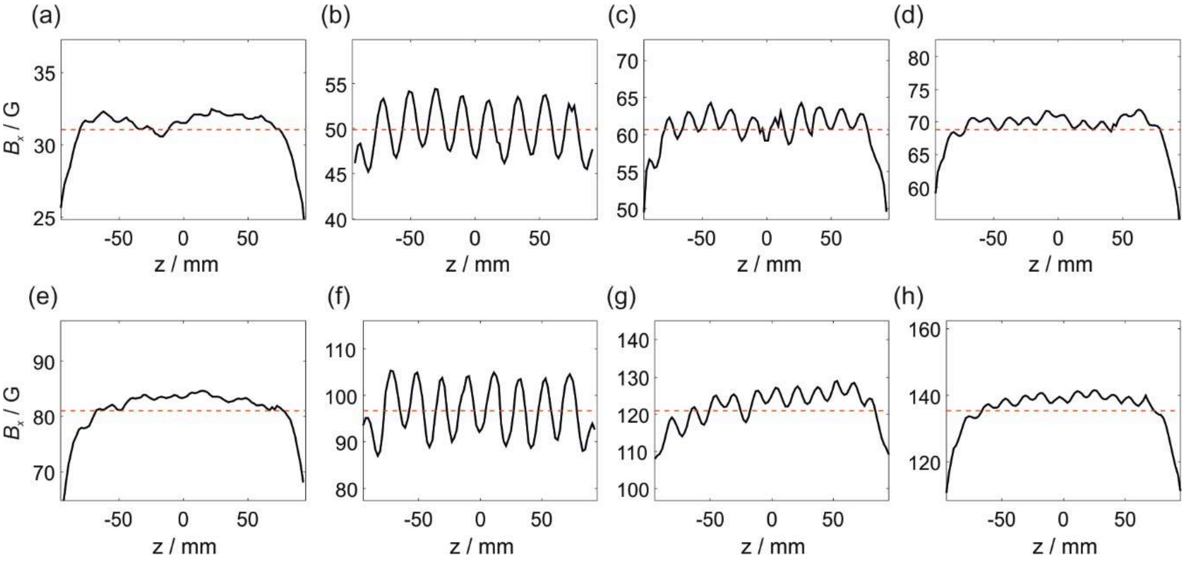

Figure S3 Magnetic field profiles of the 8 hand-held magnet arrays in Figure S1 (described by the parameters in Table 2). The profiles were measure along the central axis of the cylinder from the middle of the first ring of magnets to the middle of the final ring of magnets. The final three magnet arrays (f-h) use the same design parameters as (b-d) but with 8 magnets instead of 4 in each magnet ring and therefore the total field is approximately doubled.

Table S1 SABRE enhancement factors for 10 repeated experiments carried out with the three methods for generating the PTF. The PTF was approximately 60 G in all cases.

Method		SABRE enhancement factor			
		ortho ^{1}H	*meta* ^{1}H	*methyl* ^{1}H	Total
Flow system (Electromagnet)	1	-1235	-633	-385	-699
	2	-1203	-616	-375	-680
	3	-1199	-614	-374	-678
	4	-1360	-702	-424	-771
	5	-1245	-638	-386	-704
	6	-1310	-671	-407	-741
	7	-1196	-607	-367	-672
	8	-1294	-661	-398	-729
	9	-1315	-674	-407	-742
	10	-1270	-648	-394	-717
	Average	-1263	-646	-392	-713
	Std. Dev.	4.5%	4.7%	4.6%	4.5%
Manual shaking (stray field)	1	-5261	-2815	-1529	-2963
	2	-6668	-3500	-1845	-3696
	3	-5728	-3455	-1686	-3346
	4	-6901	-3848	-2028	-3940
	5	-5742	-2363	-1555	-2982
	6	-6901	-2657	-1811	-3507
	7	-6776	-2933	-1857	-3570
	8	-7334	-2710	-1901	-3685
	9	-6754	-3413	-1964	-3747
	10	-6307	-3402	-1845	-3565
	Average	-6437	-3110	-1802	-3500
	Std. Dev.	10.2%	15.3%	9.1%	9.1%
Manual shaking (hand-held array)	1	-7581	-4772	-2022	-4396
	2	-7838	-4686	-2426	-4618
	3	-7281	-5109	-1558	-4208
	4	-7888	-4794	-2432	-4666
	5	-7915	-5016	-2309	-4684
	6	-7285	-4396	-2270	-4310
	7	-8171	-5159	-2050	-4687
	8	-8465	-4750	-2206	-4721
	9	-7491	-4658	-1064	-3927
	10	-7483	-4429	-2433	-4446
	Average	-7740	-4777	-2077	-4466
	Std. Dev.	5.0%	5.4%	21.4%	5.8%

Table S2. Raw SABRE enhancement factors and normalised enhancement factors obtained from manual shaking SABRE experiments with the 8 different hand-held magnetic field arrays. Enhancement factors are the average of three repeat measurements. Normalised enhancement factors are calculated relative to the maximum enhancement of the *ortho* ^{1}H resonance in a PTF of ~ 60G.

PTF / G	Enhancement factor			Normalised enhancement factor		
	ortho ^{1}H	*meta* ^{1}H	*methyl* ^{1}H	*ortho* ^{1}H	*meta* ^{1}H	*methyl* ^{1}H
31.1	-4824	-634	-1000	0.5984	0.0786	0.1240
49.9	-7045	-3265	-2077	0.8738	0.4050	0.2576
60.6	-8062	-4725	-2393	1.0000	0.5860	0.2969
68.8	-7694	-4746	-2255	0.9543	0.5887	0.2797
81.0	-5961	-3645	-2018	0.7393	0.4521	0.2503
96.7	-4159	-2538	-1373	0.5158	0.3148	0.1703
120.9	-2728	-1217	-785	0.3384	0.1510	0.0973
135.4	-2343	5.98	-464	0.2906	-0.0007	0.0575

Table S3. Raw SABRE enhancement factors and normalised enhancement factors obtained from SABRE experiments acquired with an automated flow system and a range of PTF values. Normalised enhancement factors are calculated relative to the maximum enhancement of the *ortho* ^{1}H resonance in a PTF of ~ 60G.

PTF / G	Enhancement factor			Normalised enhancement factor		
	ortho ^{1}H	*meta* ^{1}H	*methyl* ^{1}H	*ortho* ^{1}H	*meta* ^{1}H	*methyl* ^{1}H
0	-278	247	5.77	0.2482	-0.2205	-0.0052
10	-489	222	-35.1	0.4373	-0.1987	0.0314
20	-696	114	-99.2	0.6221	-0.1023	0.0887
30	-764	-29.0	-158	0.6830	0.0259	0.1413
40	-817	-177	-214	0.7303	0.1580	0.1913
50	-1014	-394	-314	0.9068	0.3524	0.2805
60	-1118	-560	-380	1.0000	0.5010	0.3400
70	-1065	-561	-366	0.9519	0.5016	0.3268
80	-1033	-554	-365	0.9237	0.4957	0.3259
90	-843	-461	-302	0.7540	0.4122	0.2697
100	-686	-408	-252	0.6133	0.3650	0.2253
110	-587	-430	-230	0.5245	0.3847	0.2054
120	-386	-308	-147	0.3454	0.2753	0.1318
130	-411	-237	-144	0.3672	0.2121	0.1288
140	-362	-98.1	-98.1	0.3241	0.0877	0.0877
